# Photoelectrochemical Stability under Anodic and Cathodic Conditions of Meso-Tetra-(4-Sulfonatophenyl)-Porphyrinato Cobalt (II) Immobilized in Polypyrrole Thin Films

**DOI:** 10.3390/polym13040657

**Published:** 2021-02-23

**Authors:** Jhon Puerres, Mauro Díaz, John Hurtado, Pablo Ortiz, María T. Cortés

**Affiliations:** 1 Chemistry Department, Universidad de los Andes, Bogotá D.C. 111711, Colombia; jd.puerres@uniandes.edu.co (J.P.); ma.diazm@uniandes.edu.co (M.D.); jj.hurtado@uniandes.edu.co (J.H.); 2 Chemical Engineering Department, Universidad de los Andes, Bogotá D.C. 111711, Colombia; portiz@uniandes.edu.co

**Keywords:** photoelectrochemistry, polypyrrole, porphyrin, stability, thin films

## Abstract

Cobalt porphyrins have emerged as promising catalysts for electrochemical and photoelectrochemical applications because of their good performance, low cost and the abundance of cobalt in the earth. Herein, a negatively charged porphyrin meso-tetra-(4-sulfonatophenyl)-porphin (TPPS) was immobilized in polypyrrole (PPy) during the electro-polymerization, and then it was metallized with cobalt to obtain meso-tetra-(4-sulfonatophenyl)-porphyrinato cobalt (II) (CoTPPS) as a dopant in PPy. The coatings were evaluated as photoelectrodes towards thiosulfate oxidation and oxygen reduction. For comparison purposes, the photoelectrochemical behavior of ClO_4_^−^-doped polypyrrole films was also evaluated. Characterizations by chronoamperometry, UV-Vis spectroscopy and Raman spectroscopy showed that polypyrrole is stable under anodic and cathodic conditions, but CoTPPS and TPPS immobilized in PPy are degraded during the anodic process. Thus, decreases in photocurrent of up to 87% and 97% for CoTPPS-doped PPy and TPPS-doped PPy were observed after a 30-min chronoamperometry test. On the other hand, good stability of CoTPPS and TPPS immobilized in PPy was observed during photoelectrochemical oxygen reduction, which was reflected in almost constant photocurrents obtained by chronoamperometry. These findings are relevant to understanding the role of CoTPPS as a catalyst or pre-catalyst in photoelectrochemical applications such as water splitting. In addition, these results could pave the way for further research to include CoTPPS-doped PPy in the design of novel photocathodes.

## 1. Introduction

Water oxidation and oxygen reduction are one of the most important and challenging reactions in energy conversion. In both cases, photoelectrochemistry has emerged as an attractive alternative that makes use of solar radiation to boost the electrochemical process [1,2,3]. In photoelectrochemical water splitting, the commercial viability of the route is currently limited because of the low efficiency and/or poor stability of the photoelectrodes, which have traditionally been composed of inorganic semiconductors such as TiO_2_, ZnO, WO_3_, Fe_2_O_3_ and CdS [4,5]. With respect to the photoelectrochemical oxygen reduction reaction, it has typically been studied using thiophene-derived polymers and immobilized photosensitizers such as phthalocyanines, but some issues about photocurrent stabilities have been reported [6,7,8].

The search for better performances in electrochemical and photoelectrochemical processes has led to the evaluation of alternative compounds such as porphyrins [9,10]. When using these substances as electrocatalysts, a great advantage is the variety of transition metals that can be bonded to their rings. Even so, photoelectrochemical investigations with these compounds are currently scarce. In this field, an important point to consider about the use of porphyrins is their immobilization on conductive and semiconductor electrodes. Surface impregnation methods have been commonly used, but these methods can lead to low stabilities due to the desorption of porphyrins from the electrodes.

P. Wei et al. reported the use of Ni-meso-tetra(4-carboxyphenyl)porphyrin (NiTCPP) as a water oxidation catalyst (WOC) incorporated by soaking on the system WO_3_/TiO_2_. In this heterojunction the efficiency of hole injection in the electrolyte increased 41% compared with the heterojunction without the porphyrin. The authors reported the loss of the signals of the porphyrin (observed by UV-Vis spectroscopy) after 1 h of photolysis. This was attributed to the catalyst desorption from the photoanode [11]. Similarly, B. Liu et al. designed a functionalized photoelectrode by soaking BiVO_4_/Al_2_O_3_ in a Co-meso-tetra(4-carboxyphenyl)porphyrin (CoTCPP) solution. In this case, a two-fold enhancement in photocurrent at 1.23 V vs. RHE was achieved in comparison with the yield of BiVO_4_/Al_2_O_3_ [12].

Although there are relatively few reports of metal porphyrins in photoelectrochemical water splitting, a higher amount of papers have addressed the water oxidation process with porphyrins without the use of light [13,14,15,16]. With this last approach, some studies have shown that metal porphyrins are susceptible to degradation under anodic conditions. D. Hötger et al. reported that Fe-meso-tetra(4-pyridyl)porphyrin with Co-adsorbed cobalt on gold was degraded and Co/Fe(oxyhydr)oxides were formed as new catalysts available to carry out the oxygen evolution reaction (OER) [17]. Similarly, Q. Daniel et al. suggested the formation of thin films of CoO_X_ catalyst due to the degradation of three different cobalt porphyrins deposited on fluorine-doped tin oxide (FTO) [18], and T. Nakazono et al. evidenced that different cobalt porphyrins working as homogeneous catalysts for water splitting were prone to oxidative cleavage, but cobalt remained bounded to the oxidation products [19].

In photoelectrochemical oxygen reduction, some studies have shown the potential of porphyrins to carry out the reaction. In these systems (as in water splitting) porphyrins with carboxyl groups are frequently used to improve the attachment of the porphyrins to the electrode surfaces. D. H. Apaydin et al. coated TiO_2_ nanotubes with 5-(4-carboxy-phenyl)-10,15,20-triphenylporphyrinato copper(II) (CuTPP–COOH) and they achieved a photocurrent of 9 μA/cm^2^ at −0.3 V vs. NHE using only visible light (λ > 395 nm) [20]. In the same way, O. Jung et al. reported the sensitization of NiO with meso-tetra(4-carboxyphenyl)porphyrin (TCPP) by a soaking process. In this case, a photocurrent of 80 μA/cm^2^ (at 0.5 V vs. RHE) was achieved under AM 1.5 and 1 sun illumination. Since no photocurrent was observed for bare NiO, the photocurrent achieved was attributed to the TCPP porphyrin and the match between the reduction potential of TCPP and the valence band of NiO [21].

Although porphyrins substituted with carboxyl groups have shown the best photoelectrochemical performances, it has been observed that the interactions between the semiconductor surface and the carboxyl groups are not strong enough to ensure good attachment. In this way, alternative strategies such as the entrapment of charged porphyrins in conductive polymers are very attractive for the development of novel photoelectrodes.

Herein, a negatively charged porphyrin meso-tetra-(4-sulfonatophenyl)-porphin (TPPS) was immobilized in polypyrrole (PPy) during the electro-polymerization process, then it was metallized with cobalt to obtain meso-tetra-(4-sulfonatophenyl)-porphyrinato cobalt (II) (CoTPPS) as a dopant in PPy. For comparison purposes, ClO_4_^−^-doped polypyrrole films were also synthesized. Since porphyrins have gained attention in photoelectrochemistry, we evaluated for the first time the photoelectrochemical stabilities of CoTPPS and TPPS entrapped in PPy under anodic and cathodic conditions to perform thiosulfate oxidation and oxygen reduction, respectively. Polypyrrole was selected because of its low cost, good processability, ease of synthesis and good performance as catalyst support [22,23,24]. The results showed that TPPS and CoTPPS are susceptible to degradation under anodic conditions and that the degradation rate is dependent on the thickness of the coating and the dopant nature in PPy. Differently, a good stability of the materials was observed during photoelectrochemical oxygen reduction. These findings are valuable for the design of photoelectrodes made of porphyrins immobilized within conducting polymers, they also pave the way for further research to exploit the potential of this material, CoTPPS-doped PPy, as a photocathode.

## 2. Materials and Methods

### 2.1. Materials

FTO substrates 25 mm × 12.5 mm (Ossila, TEC 8, Sheffield, UK were cleaned for 3 min using 10% *w*/*w* NaOH (Carlo Erba, ≥97%, Val de Reuil, France) solution at 55 °C, after that, the substrates were sonicated twice in ultrapure water for 15 min. Pyrrole (Sigma Aldrich, 98%, Steinheim am Albuch, Germany) was distilled and stored under a nitrogen atmosphere. Acetonitrile (Sigma Aldrich, gradient grade for LC, Darmstadt, Germany) was kept over molecular sieves. LiClO_4_ (Sigma Aldrich, ≥95%, St. Louis, MI, USA) and (CH_3_COO)_2_Co.4H_2_O (Sigma Aldrich, ≥98%, St. Louis, MI, USA) were used without further purification.

The TPPS synthesis was adapted from a previous report [25]. Briefly, the mixture of 5,10,15,20-tetraphenylporphyrin (0.2517 g) and 96% sulfuric acid (6 mL. Merck, Darmstadt, Germany) was heated at 110 °C for 24 h. The reaction mixture was cooled to room temperature and diluted with water (80 mL). The resulting mixture was neutralized with 50% NaOH (21 mL. Carlo Erba, ≥97%, Val de Reuil, France) and methanol (50 mL. Sigma Aldrich, ≥99.9%, St. Louis, MI, USA) was added to precipitate the formed sodium sulphate. After filtration and washing the solid with methanol, the filtrate was diluted with methanol (150 mL) to precipitate more Na_2_SO_4_. The last process was repeated two more times and the resultant solution was concentrated to give a solid product with a green color. ^1^H-NMR (400 MHz, DMSO-d6) δ 8.86 (s, 8H, py), 8.20 (m, 8H, Ph−CH), 8.06 (m, 8H, Ph−CH), −2.94 (s, 2H, −NH) (Supplementary Materials Figure S1). ^13^C-NMR (100 MHz; DMSO-d6) δ 147.79, 141.33, 133.77, 124.24, 119.74 (Figure S2). UV-Vis (H_2_O, λ [nm]): 414, 516, 553, 581, 635 (Figure S3).

### 2.2. Electrochemical Synthesis of Coatings

**Figure 1:**
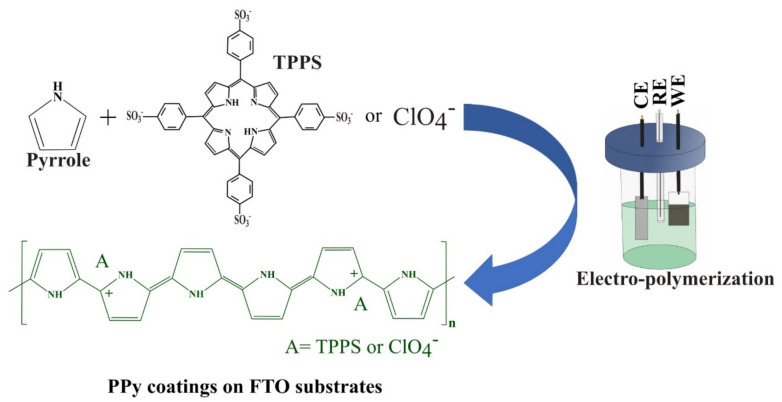
Schematic illustration of the electrochemical synthesis of meso-tetra-(4-sulfonatophenyl)-porphin (TPPS)-doped polypyrrole (PPy) and ClO_4_^−^-doped PPy. (CE: counter-electrode, RE: reference electrode, WE: working electrode: fluorine doped tin oxide (FTO)).

Pyrrole electro-polymerizations were carried out in a conventional three-electrode cell by using FTO substrates as working electrodes (WE), a platinum foil as counter-electrode (CE) and an Ag/AgCl electrode as reference electrode (RE) (Figure 1). The thickness of the coatings was controlled by the supplied electric charge. For TPPS-doped PPy, the electrosynthesis was accomplished by a galvanostatic signal (0.02 mA/cm^2^) using a precursor solution composed of 0.1 M pyrrole and 0.1 mM TPPS in deionized water. For ClO_4_^−^-doped PPy the electrosynthesis was accomplished under 0.5 mA/cm^2^ using a solution composed of 0.25 M pyrrole and 0.5 M LiClO_4_ in acetonitrile +2% *w*/*w* H_2_O. For the obtention of CoTPPS-doped PPy a previously reported route was used [26]. In this way, Co^2+^ ions were introduced into the porphyrin by immersion (10 min) of TPPS-doped PPy in aqueous 0.1 M cobalt acetate solution heated to 90 °C.

### 2.3. Characterizations

The morphology of PPy coatings was examined by scanning electron microscopy (SEM) using TESCAN LYRA3 equipment (Brno–Kohoutovice, Czech Republic). Photoelectrochemical characterizations were performed in 0.1 M Na_2_S_2_O_3_ and 0.1 M Na_2_SO_4_ for anodic and cathodic polarizations, respectively. For illumination (100 mW/cm^2^), a solar simulator ABET technologies 10500 (Milford, CT, USA) was used. UV-Vis spectra were measured with an Analytik Jena SPECORD 50 PLUS spectrophotometer (Jena, Germany). Raman spectra were taken using a 532 nm laser in a HORIBA Scientific XploRA equipment (Kyoto, Japan). IR spectra were obtained with an IRTracer-100 FTIR Shimadzu spectrophotometer (Kyoto, Japan).

## 3. Results and Discussion

### 3.1. Synthesis of Coatings and Morphological Characterization

The electrochemical synthesis of ClO_4_^−^-doped polypyrrole (PPy-ClO_4_) and TPPS-doped polypyrrole (PPy-L) were carried out in both cases supplying 42, 31 and 21 mC/cm^2^. Since the thickness of the coatings is directly related to the electric charge supplied, thicknesses of 87 nm, 64 nm and 43 nm were calculated, respectively [27].

**Figure 2:**
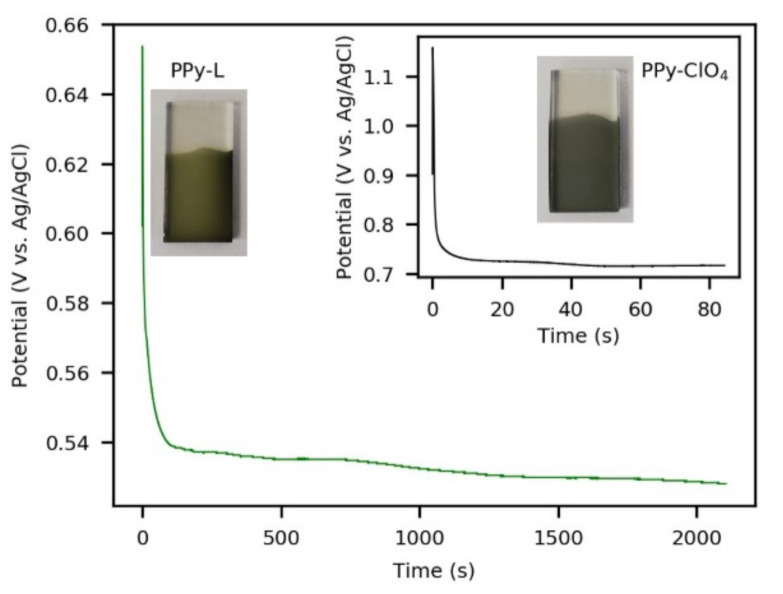
Galvanostatic polymerizations of ClO_4_^−^-doped polypyrrole (PPy-ClO_4_) and TPPS-doped polypyrrole (PPy-L). In both cases, the electric charge supplied was 42 mC/cm^2^.

The galvanostatic conditions that were used produced homogeneous polypyrrole coatings on FTO substrates. Moreover, the potentials reached during the growth of the films were low enough to ensure that polypyrrole over-oxidation did not occur (Figure 2). This potential was lower for PPy-L than for PPy-ClO_4_, suggesting more favorable conditions for electro-polymerization.

After the synthesis of PPy-L, the insertion of cobalt ions in the porphyrin was carried out to obtain CoTPPS-doped polypyrrole (PPy-C). This process did not lead to visible changes in the coatings. However, the IR spectra of the composite material showed some changes with the incorporation of cobalt (Figure S4). In the metal-free material, two absorption bands were observed in 1035 and 963 cm^−1^ wavenumbers, but these signals disappeared after metalation and a new absorption band was appreciated in 957 cm^−1^. This metal-dependent band has been previously assigned for other porphyrins as an in-plane porphyrin deformation mode [28,29]. As a complement, the presence of cobalt in PPy-C was confirmed by energy-dispersive X-ray spectroscopy (EDS) (Figure S5).

**Figure 3:**
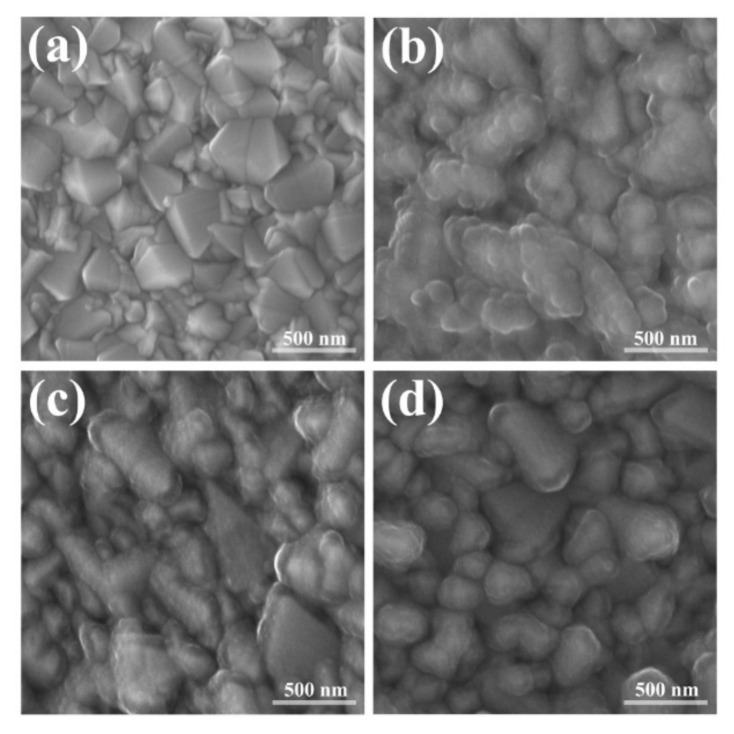
SEM images of (**a**) bare FTO, (**b**) PPy-ClO_4_, (**c**) PPy-L, (**d**) PPy-C. 42 mC/cm^2^ of electric charge supplied during polymerizations.

On the other hand, the morphology of the films was examined by scanning electron microscopy (Figure 3). PPy-ClO_4_ presented a structured shape with many and small globules on the surface (globular topology), although the shape of the FTO grains was still identified. In the case of PPy-C and PPy-L, both had a morphology with a planar tendency in which the coatings followed the shape of the FTO surface. In all cases, no uncovered areas of FTO were observed.

### 3.2. Anodic Photoresponse

**Figure 4:**
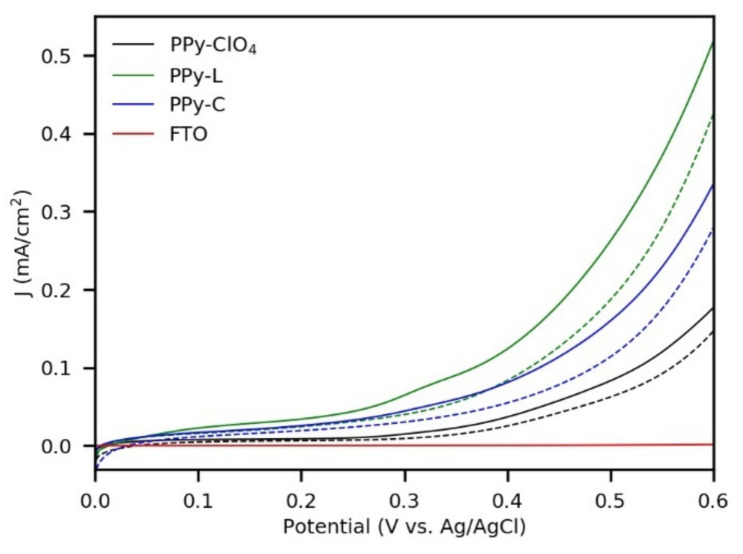
Linear sweep voltammetry (LSV) curves of bare FTO and PPy-films synthesized with 42 mC/cm^2^, under illumination (solid lines) and without illumination (dashed lines) in 0.1 M Na_2_S_2_O_3._

The photoelectrochemical performance of the composite materials under anodic conditions was examined by linear sweep voltammetry (LSV) and chronoamperometry measurements. Figure 4 depicts the LSV curves obtained with and without the illumination of the synthesized films with 42 mC/cm^2^. It can be observed that the bare FTO presented current densities near zero during the potential sweep. In contrast, all the coatings showed responses with and without illumination. Based on this, it can be suggested that these polymeric films have catalytic activity towards thiosulfate oxidation, while their photocatalytic properties were evidenced in higher current densities when irradiation was used.

With respect to the magnitude of the current densities, it had the following order from highest to lowest: PPy-L, PPy-C and PPy-ClO_4_. This trend was also observed in the coatings obtained with 31 mC/cm^2^ (Figure S6A), and in general, no noticeable differences were found between the 42 mC/cm^2^ and 31 mC/cm^2^ films. On the other hand, for the coatings deposited with 21 mC/cm^2^, it was only possible to obtain LSV curves with and without illumination for PPy-C and PPy-ClO_4_. In the case of PPy-L, it degrades rapidly and each successive LSV exhibited a curve with lower current densities (Figure S6B).

**Figure 5:**
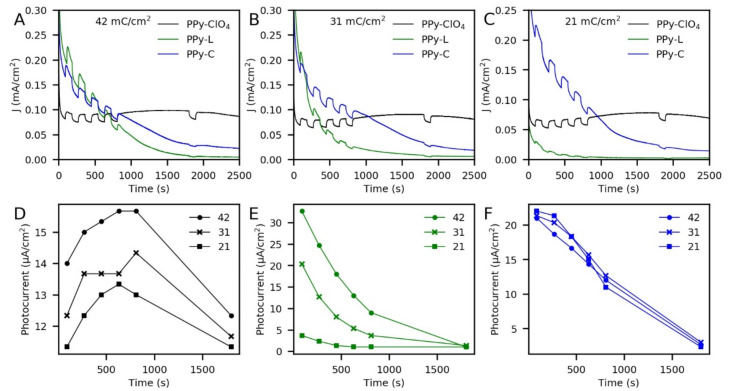
Chopped chronoamperometry of PPy-films synthesized with electric charges of: (**A**) 42 mC/cm^2^, (**B**) 31 mC/cm^2^ and (**C**) 21 mC/cm^2^. Photocurrent stability and its relation with the electric charge of polymerization for (**D**) PPy-ClO_4_, (**E**) PPy-L and (**F**) PPy-C. 0.1 M Na_2_S_2_O_3_ as electrolyte, 0.6 V vs. Ag/AgCl as applied potential.

The stability and magnitude of the photocurrents according to the dopant and thickness of the films was evaluated by chronoamperometry (at 0.6 V vs. Ag/AgCl) using chopped light. For this purpose, six changes from darkness to illumination were distributed during the characterization as can be seen in Figure 5. PPy-ClO_4_ had the highest stability of current and photocurrent regardless of the thickness of the coating (Figure 5D). This was different in the case of PPy-L (Figure 5E) and PPy-C (Figure 5F) since a progressive decrease in current and photocurrent was appreciated in both polymers, although for PPy-C the decrease was less pronounced in the thicker films. Moreover, PPy-ClO_4_ and PPy-L showed a clear relationship between thickness and photocurrent, while PPy-C exhibited no significant changes in photocurrent according to thickness. PPy-L started with the highest photocurrent of all systems (~33 μA/cm^2^) when an electric charge of 42 mC/cm^2^ was used. However, it decreased rapidly as the thickness of the coating was reduced, so that ~4 μA/cm^2^ was obtained using 21 mC/cm^2^. On the other hand, PPy-C had a slower loss of stability than PPy-L as shown by the behavior of photocurrents over time. This indicates that the presence of the cobalt complex changes the photoelectrochemical properties of the PPy coatings. However, if the thickness of the coating is related to the amount of complex entrapped, it follows that this amount does not noticeably influence the photoresponse of the electrode. In the case of the dark currents observed during all the characterizations by chronoamperometry, they can be attributed to the catalytic activity of the films to carry out the reaction. However, in the case of PPy-L and PPy-C, the dark currents could be associated with their degradation process.

The differences in stability and photoresponses between PPy-C and PPy-L, despite their similar morphologies (Figure 3), suggest that the photoelectrochemical performance depends mainly on the chemical nature of the dopant. If this were not the case, one would expect PPy-ClO_4_ to have higher currents densities by LSV, given its globular topology that confers a high surface area (Figure 3). Therefore, it appears that surface area does not noticeably influence the performance of PPy films compared to the nature of the dopant.

**Figure 6:**
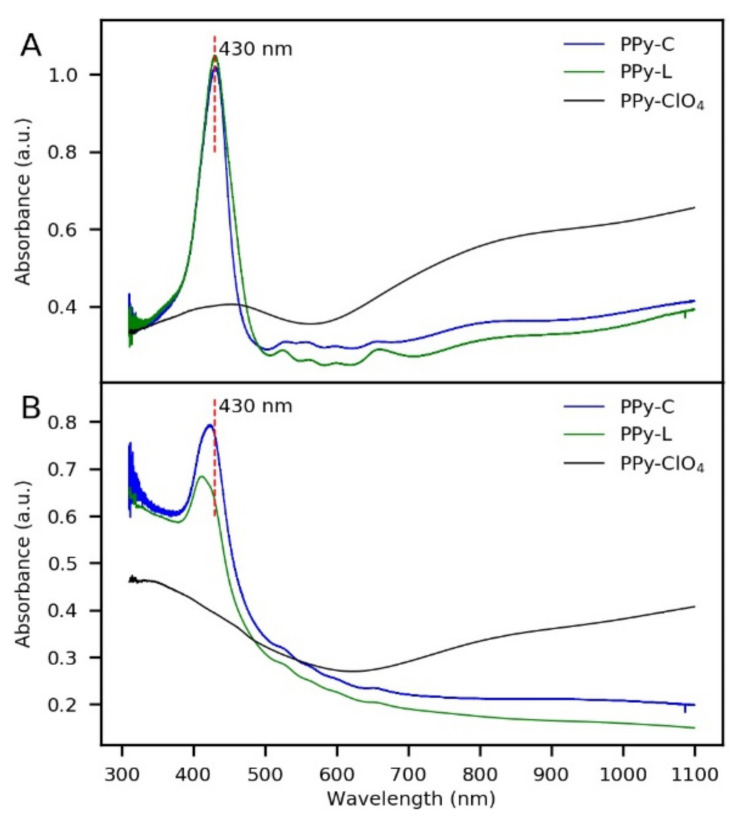
UV-Vis characterization of PPy-ClO_4_, PPy-L and PPy-C before (**A**) and after (**B**) the photoelectrochemical test under anodic conditions (light chopped chronoamperometry at 0.6 V in 0.1 M Na_2_S_2_O_3_).

In order to better understand the behavior of the photoelectrodes, characterizations by UV-Vis and RAMAN spectroscopies were made before and after the photoelectrochemical test. From UV-Vis of fresh photoelectrodes, it was possible to confirm that TPPS was immobilized in the PPy matrix due to Soret band and Q Bands of the porphyrin were clearly identified (Figure 6A). However, it was observed a red-shift of the bands compared to TPPS in aqueous solution (Table 1). A similar red-shift was reported by T. Hatano et al. and it was attributed to TPPS aggregates as dopants in PEDOT and PPy [30]. In the present case, the formation of aggregates in the polymer is unlikely since the four Q bands of the porphyrin were identified after polymerization, and it is well known that TPPS aggregates have only one Q band [31,32]. For this reason, the red-shift observed is possibly attributed to the interaction of TPPS with the polymer.

In the case of the absorption spectrum of PPy-C, no differences were observed in the Soret band position in comparison with the Soret band observed in PPy-L. Nevertheless, in PPy-C the intensities of Q bands decreased and there was an overlapping of the bands at 528 nm and 558 nm. Since CoTPPS in solution has two absorption bands at 425 nm and 538 nm [33,34], it is very likely that the method of inserting cobalt into the polymer led to a partial complexing of the total TPPS molecules in the coating. However, the amount of CoTPPS formed in PPy was enough to change the photoelectrochemical response of the system (previously observed in Figure 5). On the other hand, it is known that the photoelectrochemical performance of a photoactive material is related to its optical band gap since this determines the amount of radiation that the material can absorb. In Figure 6A is observed an absorption band at 455 nm for PPy-ClO_4_. This can be attributed to the π–π* transition of the polymeric backbone, so it is possible to calculate the optical bandgap of the material using the onset of the transition. Nevertheless, in the case of CoTPPS-doped PPy and TPPS-doped PPy it is not possible to use the UV-Vis data to determine the optical band gap since CoTPPS and TPPS have intense absorption bands that dominate the absorption spectra. For this reason, it is not possible to make a comparison between the bandgap of these materials from this technique.

With respect to the optical properties of the coatings after the photoelectrochemical test, significant changes were found as shown in Figure 6B. PPy-C and PPy-L had a decrease in the intensity of Soret bands and Q bands were barely identified. PPy-ClO_4_ also showed a decrease in the absorption of visible light, which could be attributed to de-doping during the photoelectrochemical characterization. Nevertheless, as previously observed, de-doping in PPy-ClO_4_ did not greatly affect the stability of photocurrents. On the other hand, in PPy-C and in PPy-L de-doping is more difficult because of the large size of dopants. Therefore, the lower intensity of Soret bands and the flattering of Q bands could be the result of porphyrin degradation which is consistent with the low stability of currents and photocurrents generated by PPy-C and PPy-L. Additionally, Soret bands of PPy-L and PPy-C had a blue-shift of 6 nm and 19 nm, respectively. It suggests that the degradation products of the porphyrin molecules did not leak to the electrolyte.

**Figure 7:**
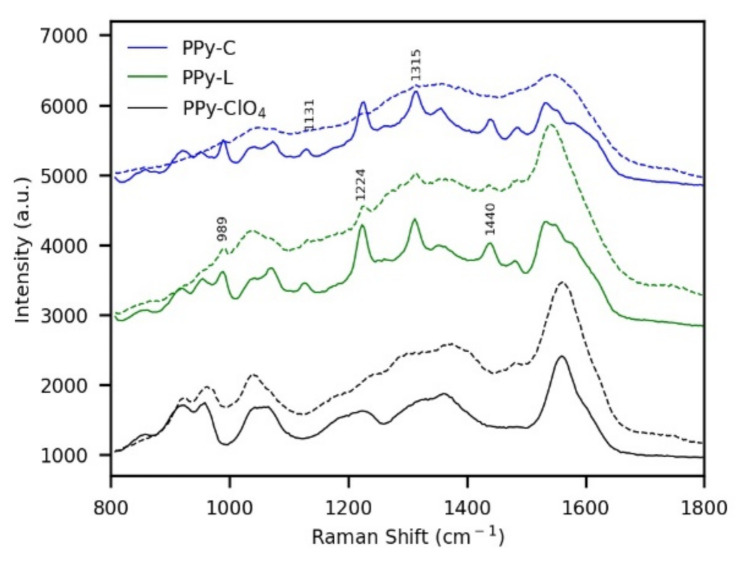
Raman characterization of PPy-ClO_4_, PPy-L and PPy-C before (continuous lines) and after (dashed lines) the photoelectrochemical test under anodic conditions (light chopped chronoamperometry at 0.6 V in 0.1 M Na_2_S_2_O_3_).

Raman spectroscopy was also used to analyze the photoelectrodes before and after the photoelectrochemical test (Figure 7). All fresh coatings showed common bands attributed to PPy in 860 cm^−1^, 922 cm^−1^, 958 cm^−1^ and 1362 cm^−1^. Additionally, in PPy-L and PPy-C some bands of porphyrin were identified. In the case of PPy-ClO_4_, 1560 cm^−1^ band and 1600 cm^−1^ shoulder are ascribed to a mixed C=C and C–C vibration of neutral PPy and oxidized PPy, respectively. The 1362 cm^−1^ band and 1330 cm^−1^ shoulder are attributed to the ring stretching mode of PPy. The overlapped bands of 1044 cm^−1^ and 1065 cm^−1^ are assigned to C–H in-plane deformation, and at 922 cm^−1^ and 958 cm^−1^ are associated with the ring deformation in bipolarons and polarons, respectively [35,36].

The signals associated with TPPS and CoTPPS immobilized in the polymer are identified in Figure 7. These signals were clearly flattened after the photoelectrochemical test, which is consistent with the degradation of the porphyrin molecules. Unlike what was found in absorption spectra, Raman spectra of PP-L and PPy-C showed practically no signals of dopants or degradation products after photoelectrochemical evaluation. However, this was probably due to the overlapping of the signals with PPy bands.

Since catalytic properties of cobalt towards electrochemical water oxidation are well known, we evaluate the oxidation potential of water with PPy-L and PPy-C after subjecting them to linear sweep voltammetry up to high anodic potentials. This in order to draw conclusions about the stability of cobalt in the polymer matrix (Figure S7). It was found that although PPy-C is degraded, cobalt entrapped in the film was enough to enhance the electrocatalytic water oxidation (Figure S7B). In this way, an approximate fifteen-fold enhancement in current density (at 2 V vs. RHE) was obtained with degraded PPy-C compared with degraded PPy-L.

### 3.3. Cathodic Photoresponse

**Figure 8:**
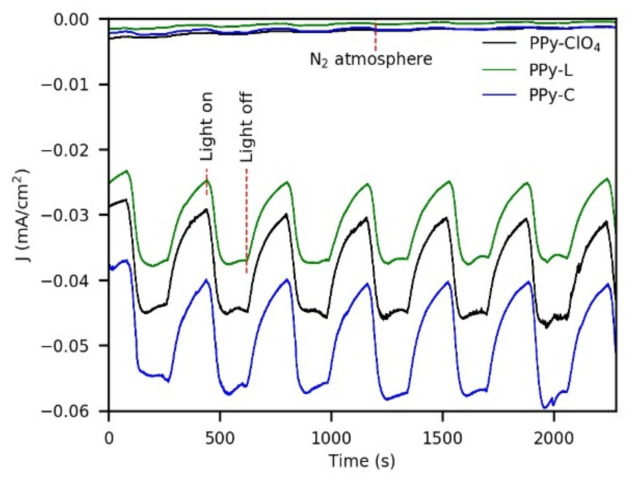
Chopped chronoamperometry of PPy-films in 0.1 M Na_2_SO_4_ at −0.4 V vs. Ag/AgCl (synthesis charge of all films was 42 mC/cm^2^). The notation “N_2_ atmosphere” indicates characterizations in deoxygenated solutions.

Chronoamperometry measurements using chopped light were performed at −0.4 V vs. Ag/AgCl in 0.1 M Na_2_SO_4_ to study the stability of the composite materials under cathodic conditions (periods of illumination and darkness lasted 3 min each one). For this purpose, photoelectrodes of PPy-ClO_4_, PPy-L and PPy-C were synthesized with 42 mC/cm^2^. Characterizations were carried out in solutions previously bubbled with N_2_ and with air for 15 min to analyze the activity of the photoelectrodes towards oxygen reduction (Figure 8). In the case of deoxygenated solutions, a nitrogen atmosphere was maintained over the solution during the test. In the characterization, all the photoelectrodes showed photocathodic currents under the air atmosphere. These photocurrents can be attributed to oxygen reduction since negligible currents and photocurrents were obtained under nitrogen atmosphere.

The photocurrents presented very small variations during the test, which is a sign of the stability of the photoelectrodes under the conditions used. The mean value of photocurrents for PPy-ClO_4_ was ~14 μA/cm^2^, for PPy-L was ~11 μA/cm^2^ and for PPy-C was ~16 μA/cm^2^. Both dark currents and light currents presented the following order in terms of magnitude: PPy-L < PPy-ClO_4_ < PPy-C. The enhanced current of PPy-C compared with PPy-L is attributed to the presence of cobalt since it is known that cobalt has a catalytic effect on oxygen reduction [37]. At this point, it is worth noting that the cathodic photocurrents obtained in this work are in the same order of magnitude as those reported in the literature for other systems based on coordination complexes or organic semiconductors (Table 2). A weakness of several of these materials remains their low stability as photoelectrodes. For example, A.S. Konev et al. studied a polymeric metal salen-type complex that produced a photocurrent of 23 μA/cm^2^ at pH 1, but the stability was low due to the tendency of these complexes to undergo hydrolysis in acid media [38]. In other cases, photoelectrodes of thin films of organic pigments of the epindolidione (EPI) and quinacridone (QNC) family have exhibited good stabilities, but their syntheses required many steps and specialized equipment [39].

In this study, we show that CoTPPS-doped PPy presents relatively high stability in its performance as a photocathode and has the great advantage of being easily synthesized. We suggest that this material, obtained with this methodology, could be conveniently used to sensitize inorganic semiconductors with fine-tuning of the coating thickness, as well as in the design of novel CoTPPS-doped PPy/organic semiconductors heterojunctions. The latter is a promising strategy to achieve large improvements in photocurrents, as reported by M. Gryszel et al. who obtained 800 μA/cm^2^ at 0 V vs. Ag/AgCl using the H_2_Pc/PTCDI/Au system and obtained negligible photocurrents using H_2_Pc and PTCDI separately [40]. Here, H_2_Pc is metal-free phthalocyanine, PTCDI is *N,N′*-dimethyl perylenetetracarboxylic bisimide and Au represents a thin gold layer on the heterojunction.

Regarding the response speed of the coatings to lighting, some differences were observed between cathodic and anodic conditions. After the illumination of the films, a photoresponse was immediately appreciated in all cases. However, under cathodic conditions, the maximum photocurrents were reached in approximately 70–80 s, but under anodic conditions, the maximum photocurrents were always reached before 35 s.

After the photoelectrochemical characterizations in the cathodic potential, no significant changes were observed in the absorption spectra of the photoelectrodes (Figure S8B). In the case of PPy-L and PPy-C, Soret bands presented a similar intensity before and after the test. Additionally, Q bands were clearly identified after the characterizations and no signals of porphyrin degradation were appreciated. This was consistent with the good stability of the photocurrents under cathodic conditions. Moreover, Raman spectroscopy was also used to study the chemical stability of the photoelectrodes (Figure S9). In this case, all the Raman bands identified on fresh materials remained after the photoelectrochemical test, which supports the results of chronoamperometry and UV-Vis measurements.

## 4. Conclusions

The cobalt porphyrin CoTPPS and its free-base porphyrin TPPS were entrapped in polypyrrole by electropolymerization. This allowed obtaining CoTPPS-doped PPy and TPPS-doped PPy with controlled thicknesses. Photoelectrochemical and spectroscopic characterizations showed that CoTPPS and TPPS in PPy degrade easily under anodic conditions, which was reflected in the poor stability of the photocurrents during oxidation of the electron donor thiosulfate (photocurrent losses of up to 87% and 97% were observed for CoTPPS-doped PPy and TPPS-doped PPy, respectively). On the other hand, it was found that CoTPPS and TPPS entrapped in PPy are stable in photoelectrochemical oxygen reduction since nearly constant photocurrents were obtained by chronoamperometry (16 μA/cm^2^ for CoTPPS-doped PPy and 11 μA/cm^2^ for TPPS-doped PPy). Our results show the potential of these composite coatings in the electrocatalysis of light-assisted cathodic reactions. They also suggest that these materials are not suitable in anodic reactions, such as photoelectrochemical oxidation of water. In the case of ClO_4_^−^-doped PPy, dark currents and photocurrents showed stability under anodic and cathodic conditions, which reinforces the strength of polypyrrole as a matrix material in the development of photoelectrodes.

## Figures and Tables

**Table 1:** Absorption bands of TPPS and meso-tetra-(4-sulfonatophenyl)-porphyrinato cobalt (II) (CoTPPS).

System	Soret Band (nm)	Q Bands (nm)
TPPS in solution [31,32]	413	515. 552, 580, 634
TPPS in solution (this work)	414	516, 553, 581, 635
TPPS immobilized in PPy	430	524, 561, 598, 660
CoTPPS in solution [33,34]	425	425, 538
CoTPPS immobilized in PPy	431	(528 and 558 overlapped), 598, 658

**Table 2:** Comparison of photocurrent densities (J_Ph_) in photoelectrochemical oxygen reduction. Pc-D refers to phthalocyanine derivative.

Photoelectrode	Bias	Electrolyte	J_Ph_ (μA/cm^2^)	Reference
CoTPPS-doped PPy (PPy-C)	−0.4 V vs. Ag/AgCl	0.1 M Na_2_SO_4_	16	This work
P3HT treated with oxygen plasma (3-7 P3HT-PS)	0 V vs. Ag/AgCl	H_2_SO_4_ pH 1	~15	[6]
Thin film based on functionalized thiophene (Film(dimer-L^2^))	0 V vs. SCE	0.1 M Na_2_SO_4_	26.89	[7]
Zn Pc-D and PVDF mixture on ITO (2Zn)	−0.12 V vs. NHE	0.5 M KNO_3_	15.5	[8]
Mg Pc-D and PVDF mixture on ITO (1Mg)	−0.12 V vs. NHE	0.5 M KNO_3_	9.3	[8]
Polymeric metal salen-type complex ([Ni(MeOSalen)]_n_)	0 V vs. Ag/AgCl	1 M Na_2_SO_4_ + HCl pH 1	23	[38]
Thin film of hydrogen-bonded organic pigment (EPI)	0 V vs. Ag/AgCl	0.1 M HCl	15	[39]
Thin film of biscoumarin-containing acene	0 V vs. Ag/AgCl	Na_2_SO_4_ + HCl pH 2	3	[41]
Thin film of eumelanin	0 V vs. Ag/AgCl	pH 1	8	[42]
Ru complex polymerized on ITO (Poly(RuL_3_)_1_)	0 V vs. SCE	0.1 M Na_2_SO_4_	10.7	[43]
Ru complex polymerized on ITO (Poly(RuL_3_)_2_)	0 V vs. SCE	0.1 M Na_2_SO_4_	1.36	[44]

## Data Availability

Data is contained within the article and the supplementary material.

## Supplementary Materials

The following are available online at https://www.mdpi.com/2073-4360/13/4/657/s1. Figure S1: 1H NMR spectrum of meso-tetra-(4-sulfonatophenyl)-porphin (TPPS); Figure S2: 13C NMR spectrum of meso-tetra-(4-sulfonatophenyl)-porphin (TPPS); Figure S3: UV-Vis spectrum of 0.2 μM TPPS in deionized water; Figure S4: Fourier transform infrared (FT-IR) spectra of TPPS-doped polypyrrole (PPy-L) and CoTPPS-doped polypyrrole (PPy-C); Figure S5: Energy-dispersive X-ray spectrum of PPy-C; Figure S6: LSV curves of PPy-films synthesized with (A) 31 mC/cm2 and (B) 21 mC/cm2. under illumination (solid lines) and without illumination (dashed lines) in 0.1 M Na2S2O3; Figure S7: Linear sweep voltammetry of PPy-L and PPy-C in phosphate buffer pH 7.0. Scan rate 5 mV/s. (A) PPy over-oxidation during the first LSV. (B) Water oxidation after the degradation (over-oxidation) of polypyrrole; Figure S8: UV-Vis characterization of PPy-ClO4, PPy-L and PPy-C before (A) and after (B) photoelectrochemical test under cathodic conditions; Figure S9: Raman characterization of PPy-ClO_4_, PPy-L and PPy-C before (continuous lines) and after (dashed lines) photoelectrochemical test under cathodic conditions.
