# Supplementary material for: Photoelectrochemical Stability under Anodic and Cathodic Conditions of Meso-Tetra-(4-Sulfonatophenyl)-Porphyrinato Cobalt (II) Immobilized in Polypyrrole Thin Films
Source: Polymers (Basel). 2021 Feb 23;13(4):657. doi: 10.3390/polym13040657 (PMC7926596; doi:10.3390/polym13040657)
Supplement: Supplementary file 1 [file polymers-13-00657-s001.pdf]

polymers

MDPI

Article

Supplementary Materials

Photoelectrochemical Stability under Anodic and Cathodic Conditions of meso-tetra-(4-sulfonatophenyl)-porphyrinato Cobalt (II) Immobilized in Polypyrrole Thin Films

Jhon Puerres [1], Mauro Díaz [1], John Hurtado [1], Pablo Ortiz [2] and María T. Cortés [1,*]

[1] Chemistry Department, Universidad de los Andes, Bogotá D.C. 111711, Colombia; jd.puerres@uniandes.edu.co (J.P.); ma.diazm@uniandes.edu.co (M.D.); jj.hurtado@uniandes.edu.co (J.H.)
[2] Chemical Engineering Department, Universidad de los Andes, Bogotá D.C. 111711, Colombia; portiz@uniandes.edu.co
* Correspondence: marcorte@uniandes.edu.co; Tel.: +571 3394949-3132

Citation: Puerres, J.; Díaz, M.; Hurtado, J.; Ortiz, P.; Cortés M. T. Photoelectrochemical stability under anodic and cathodic conditions of meso-tetra-(4-sulfonatophenyl)-porphyrinato cobalt (II) immobilized in polypyrrole thin films. *Polymers* **2021**, *13*, 657. https://doi.org/10.3390/polym13040657

Received: 31 December 2020
Accepted: 26 January 2021
Published: 23 February 2021

Publisher's Note: MDPI stays neutral with regard to jurisdictional claims in published maps and institutional affiliations.

Copyright: © 2021 by the authors. Licensee MDPI, Basel, Switzerland. This article is an open access article distributed under the terms and conditions of the Creative Commons Attribution (CC BY) license (http://creativecommons.org/licenses/by/4.0/).

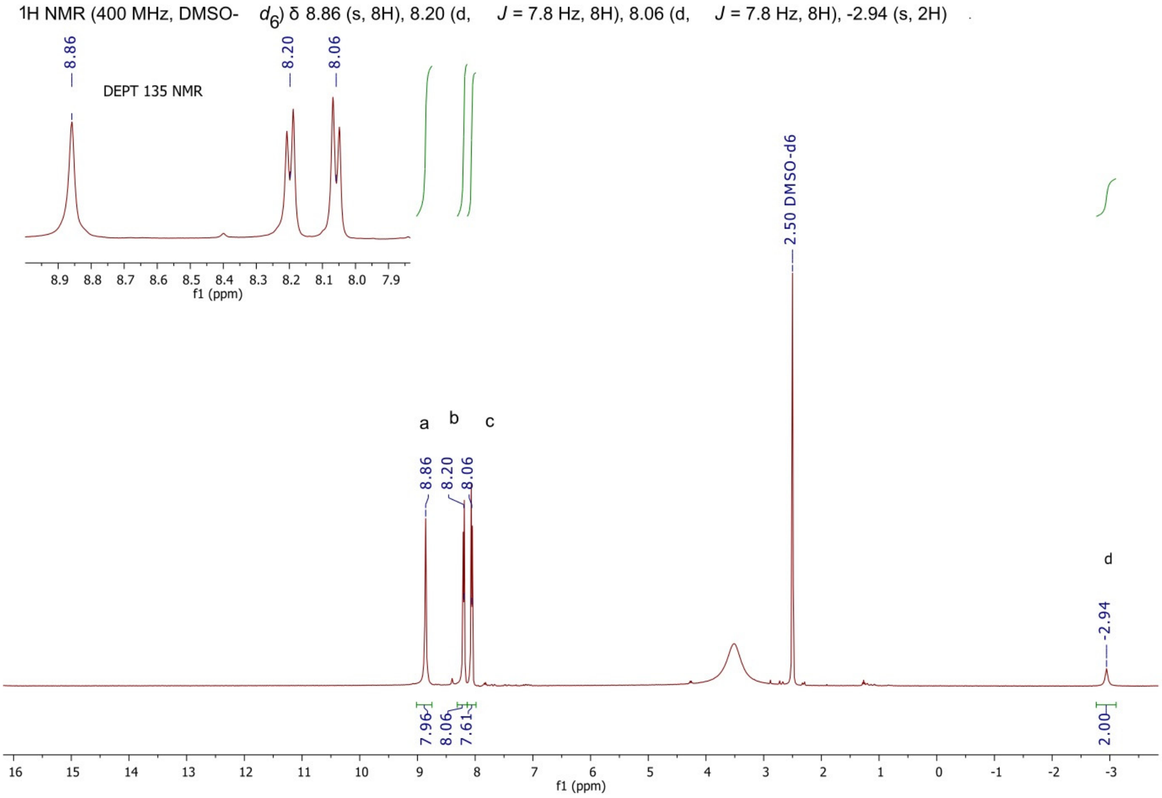

Figure S1. ^{1}H NMR spectrum of meso-tetra-(4-sulfonatophenyl)-porphin (TPPS).

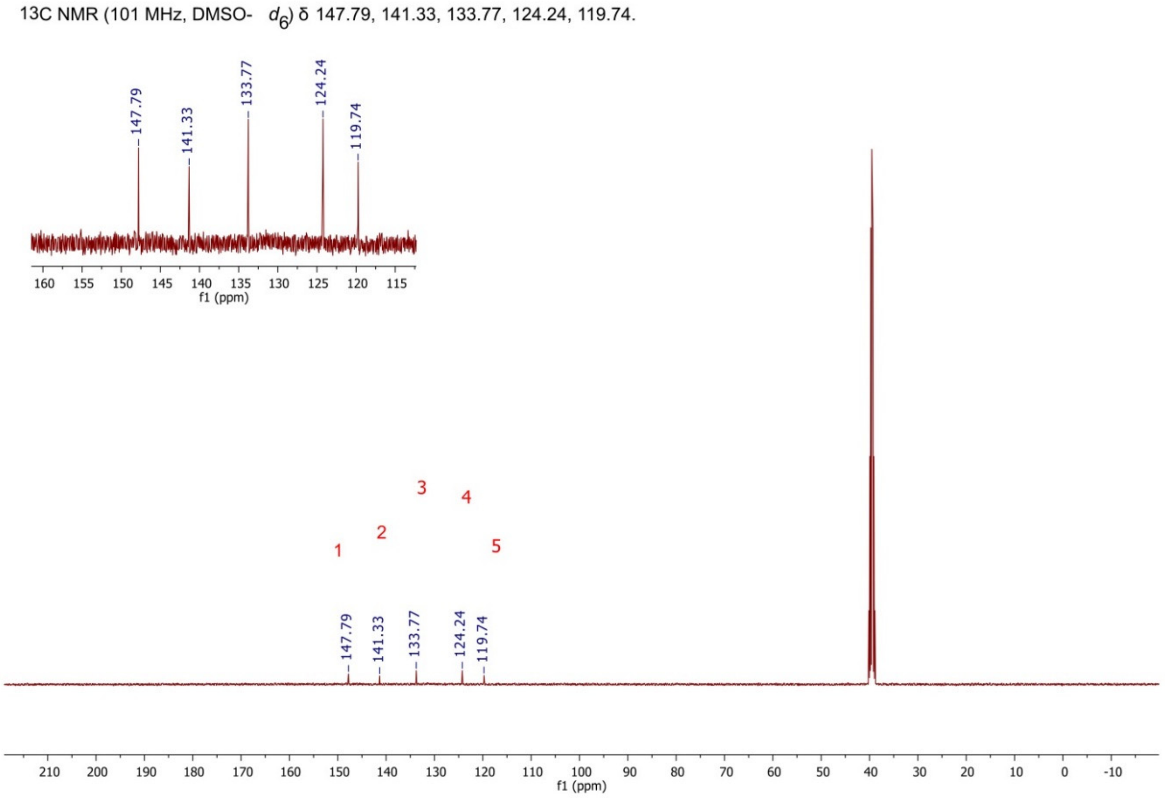

Figure S2. ^{13}C NMR spectrum of meso-tetra-(4-sulfonatophenyl)-porphin (TPPS).

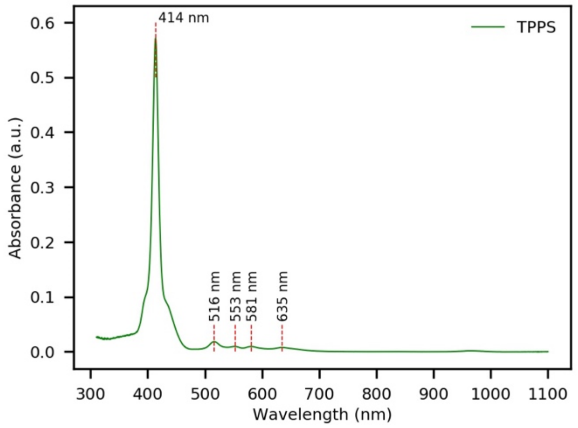

Figure S3. UV-Vis spectrum of 0.2 μM TPPS in deionized water.

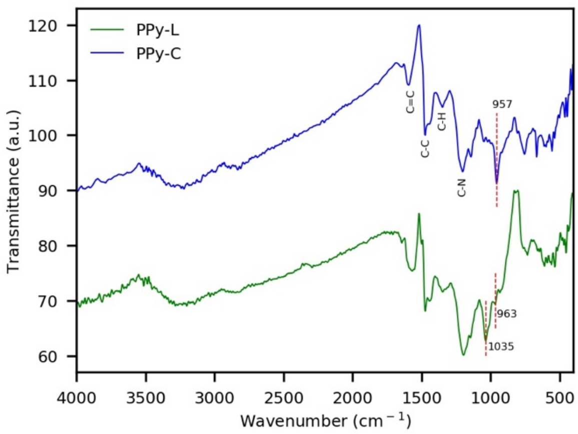

Figure S4. Fourier transform infrared (FT-IR) spectra of TPPS-doped polypyrrole (PPy-L) and CoTPPS-doped polypyrrole (PPy-C).

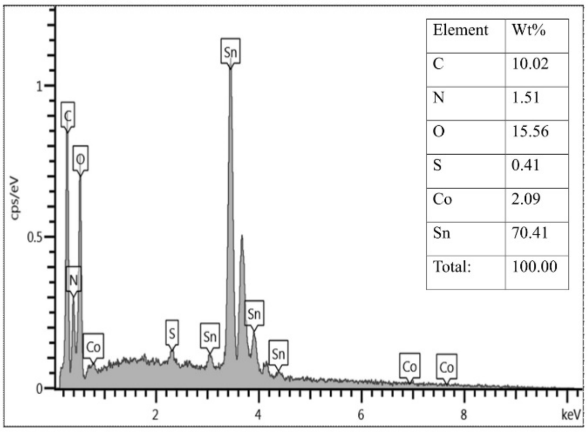

Figure S5. Energy-dispersive X-ray spectrum of PPy-C.

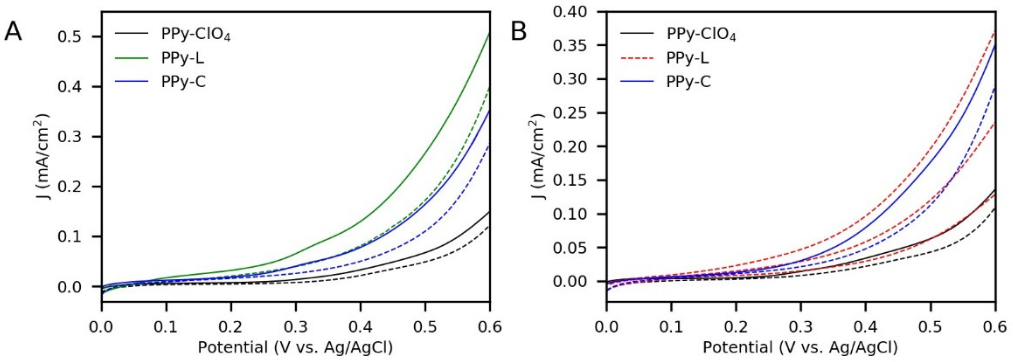

Figure S6. LSV curves of PPy-films synthesized with (**A**) 31 mC/cm² and (**B**) 21 mC/cm². under illumination (solid lines) and without illumination (dashed lines) in 0.1 M $Na_2S_2O_3$.

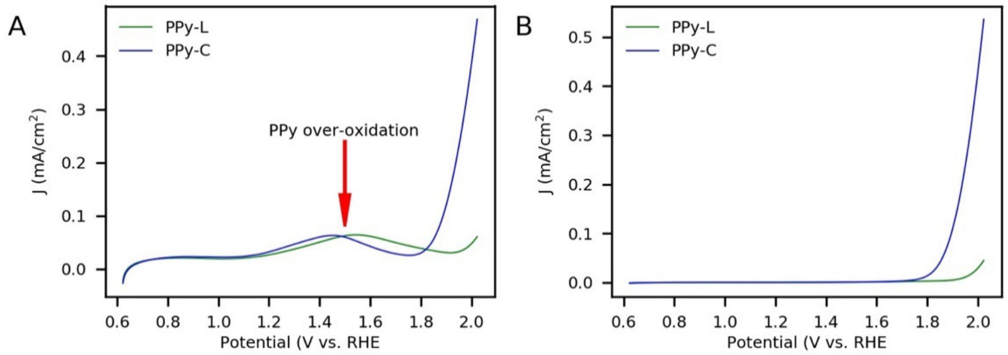

Figure S7. Linear sweep voltammetry of PPy-L and PPy-C in phosphate buffer pH 7.0. Scan rate 5 mV/s. (**A**) PPy over-oxidation during the first LSV. (**B**) Water oxidation after the degradation (over-oxidation) of polypyrrole.

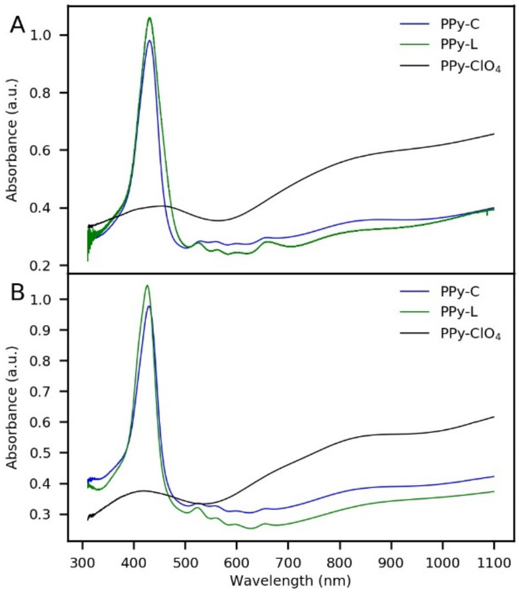

Figure S8. UV-Vis characterization of PPy-ClO$_4$, PPy-L and PPy-C before (**A**) and after (**B**) photoelectrochemical test under cathodic conditions.

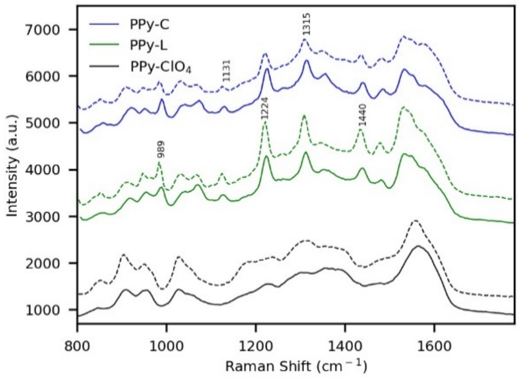

Figure S9. Raman characterization of PPy-ClO$_4$, PPy-L and PPy-C before (continuous lines) and after (dashed lines) photoelectrochemical test under cathodic conditions.
